# Non-Adaptive Defense Mechanisms and Their Relationship to Psychosomatic Disorders among a Sample of University Students

**DOI:** 10.1192/j.eurpsy.2024.872

**Published:** 2024-08-27

**Authors:** D. M. M. M. Almajali, N. S. G. Abdelrasheed

**Affiliations:** Education, dhofar University, dhofar, Oman

## Abstract

**Introduction:**

In the university stage, the student is exposed to many psychological changes, pressures, and conflicts, which makes him resort to many non-consensual psychological defense mechanisms such as (repression, justification, projection, relapse, denial, delusional illness, reverse transference, daydreaming), which causes an imbalance in the personality and its psychological functions. This may lead to cognitive and mental distortions and physiological imbalances, and the appearance of symptoms that cause psychosomatic disorders that are not due to organic physiological imbalances or bacterial diseases, but rather as a result of imbalances in the psychological functions of the ego ,Which increases the symptoms of headache, vomiting, poor digestion, irritable bowel syndrome, shortness of breath, rapid heartbeat, hormonal imbalance, facial redness, and others.

**Objectives:**

1.Identifying the degree of use of non-consensual psychological defense mechanisms among university students, and the differences in this according to the variable (gender and degree of academic achievement)

1. Revealing the correlation between the degree of use of non-consensual psychological defense mechanisms and the emergence of disturbed psychosomatic symptoms in the functions of (the respiratory system, the digestive system, the cardiac system, the muscular system, sleep disorders, and bodily disorders).

**Methods:**

The correlational analysis approach was used to study the relationship between the variables of the study. The sample consisted of 300 male and female university students. A scale for psychological defense mechanisms was constructed, and a scale for psychosomatic disorders prepared by Diop (2011) was adopted, and its psychometric properties were verified.

**Results:**

The responses in the degrees of non-consensual psychological defense mechanisms were varied, with a high degree in (justification, projection, repression, and delusional illness) and a moderate degree in (relapse, daydreaming, denial, and reverse transference). Differences appeared between males and females in favor of males, while differences in academic grades were in favor of the lowest grade. The results also showed a statistically significant correlation between psychological defense mechanisms and the appearance of psychosomatic symptoms, as it was high in disorders (respiratory system, cardiac system, muscular system, sleep disorders), and moderate in (emotional disorders and somatic disorders).

**Conclusions:**

There is a positive correlation between the degrees of use of non-consensual psychological defense mechanisms and the emergence of psychosomatic disorders, in the functions of several bodily systems and behavioral and emotional disorders.

**Disclosure of Interest:**

None Declared

